# Evaluation of airway involvement and treatment in patients with relapsing polychondritis

**DOI:** 10.1038/s41598-023-35616-4

**Published:** 2023-05-23

**Authors:** Hiroshi Handa, Seido Ooka, Jun Shimizu, Noboru Suzuki, Masamichi Mineshita

**Affiliations:** 1 Division of Respiratory Medicine, Department of Internal Medicine, St. Marianna University School of Medicine, Kawasaki City, Kanagawa Japan; 2 Division of Rheumatology and Allergology, Department of Internal Medicine, St. Marianna University School of Medicine, Kawasaki City, Kanagawa Japan; 3 Department of Immunology and Medicine, St. Marianna University School of Medicine, Kawasaki, Japan

**Keywords:** Medical research, Outcomes research, Drug therapy, Therapeutic endoscopy

## Abstract

Airway involvement in relapsing polychondritis (RP) can be debilitating and life threatening, often requiring interventional procedures. If standard therapies including systemic corticosteroid and immunosuppressive agents are ineffective, airway stenting is often required. Recently, biologics have been reported to be effective for RP, and the early administration of biologics may avoid airway stenting. To evaluate survival rates and treatment approaches, medical records of RP patients with airway involvement were reviewed. These cases were divided into the following groups: with and without malacia, stenting and non-stenting, and with and without biologics. Kaplan–Meier was used to calculate survival rates and log rank tests were used to analyze biologics groups. A total of 77 patients were enrolled. Airway stenting was performed in 13 patients, all of which developed airway malacia. The stenting group had significantly lower survival rates than the non-stenting group (*p* < 0.001). Stent-related complications were granulation tissue (85%) and mucostasis (69%). In the non-stenting group, a lower mortality rate was observed. A significantly higher survival rate was seen in patients administered biologics than without (*p* = 0.014). The early administration of biologics shows promise in preventing severe airway disorders that require airway stenting.

## Introduction

Relapsing polychondritis (RP) is a rare systemic disease that is characterized by cartilage inflammation for multiple organs. Airway involvement in RP, such as chondritis of the larynx and tracheobronchial tree, is the most critical prognosis factor. RP often results in tracheobronchomalacia (TBM), which can lead to dynamic airway collapse^1–3^ and laryngeal stenosis, that can cause choking. TBM and laryngeal stenosis can be debilitating and life threatening, often requiring interventional procedures. Therefore, the early detection of airway abnormalities is important for the prevention of airway involvement in RP.

There are several severe airway disorders that can occur, both with and without malacia, such as subglottic stenosis and tracheobronchial stenosis. When subglottic stenosis is severe, tracheostomy is needed. Invasive positive pressure ventilation (IPPV) and non-invasive positive pressure ventilation (NIPPV) are helpful to manage TBM^4,5^. If IPPV and NIPPV are ineffective due to severe tracheobronchial obstruction, interventional bronchoscopy such as airway stenting is required^1,6,7^.

Airway involvement in RP can cause airway oedema and stenosis from airway inflammation, which can result in TBM. Airway stenting is indicated for severe TBM to avoid choking. However, airway stenting has several critical complications, such as difficulty in expectoration, stent migration, and restenosis due to granulation. Furthermore, airway malacia can extend from the central airway to the peripheral airways, and the implantation of multiple metal stents may be required. Long-term stenting increases stent-related complications and the indication of airway stenting should be carefully decided.

Systemic corticosteroid and immunosuppressive agents are administered as a standard treatment for RP; however, these medications can often be ineffective for airway involvement. Recently, biologics such as infliximab and tocilizumab have been reported to be effective for RP, with the early use of biologics possibly preventing severe airway disorder^8^.

The aim of this study was to assess treatment options for RP patients with airway involvement by comparing survival rates between stenting and non-stenting groups and evaluating the efficacy of biologics.

## Results

**Figure 1:**
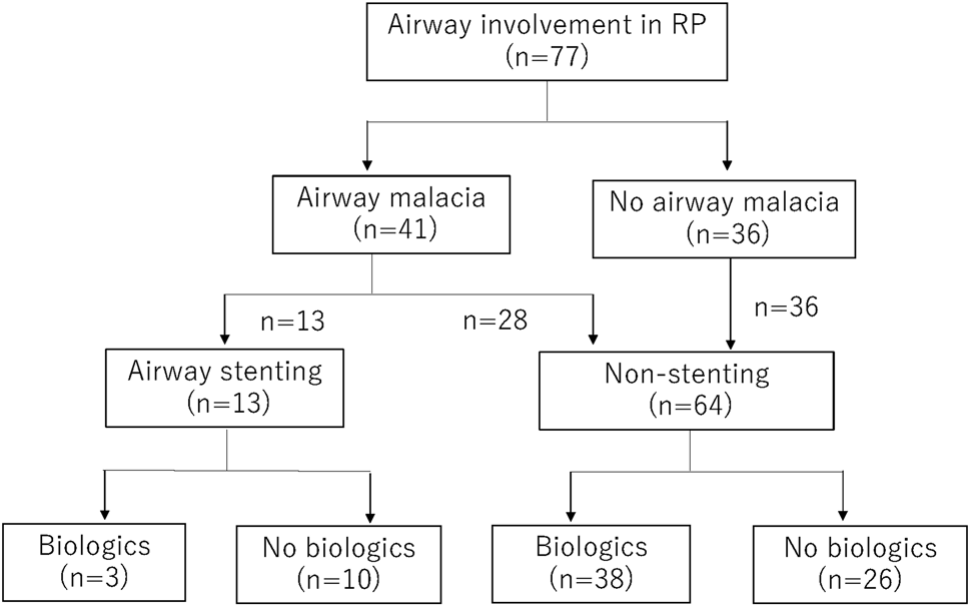
Study flow chart.

Patient characteristics are shown in Table 1. Of the 77 RP patients enrolled with airway involvement, 41 developed into airway malacia (Fig. 1), including all 13 patients from the airway stenting group. Of these 13 patients, 12 were TBM and 1 was a localized left main bronchial malacia (Table 2). In the stenting group, tracheostomy was performed in 7 patients, with 6 requiring mechanical ventilation. Two patients were administered NIPPV. Mechanical ventilation could not be performed in one patient due to an ill-fitting Montgomery T tube.

**Table 1:** Characteristics of airway involvement by RP for each group.

	Stenting (n = 13)	%	Non-stenting (n = 64)	%
Demographics				
Age [range]	66.2 [50–90]		55.7 [22–90]	
Females	8	61.5	34	53.1
Clinical characteristics				
Auricular chondritis	3	23.1	14	21.9
Nasal chondritis	2	15.4	25	39.1
Respiratory tract chondritis	13	100	64	100
Cochlear and/or vestibular dysfunction	4	30.8	23	35.9
Non-erosive inflammatory polyarthritis	4	30.8	27	42.19
Ocular inflammation	2	15.4	23	35.9
Airway malacia	13	100	28	43.8
Location of airway lesion				
Trachea	0	0	19	29.7
Trachea and both main bronchus	12	92.3	44	68.7
Left main bronchus	1	7.7	1	1.6
Medications				
Oral corticosteroid	13	100	63 (2)	98
Immunosuppressive agents	7 (4)	54	53 (6)	83
Biologics	3 (1)	23	39 (3)	57
Infliximab	2 (2)	15	29 (12)	45
Certolizumab	1 (1)	8	5 (2)	8
Golimumab	1 (1)	8	1 (0)	1
Tocilizumab	3 (1)	21	22 (8)	34
Abatacept	1 (1)	8	1 (1)	1
Rituximab	1 (1)	8	1(0)	1
Janus kinase inhibitor	1	8	0	0
Treatments				
IPPV	6	46	6	9
NIPPV	2	15	13 (1)	20
tracheostomy	7	54	18 (3)	28
Mortality	10	77	7	11
Pneumonia	3		2	
Respiratory failure	6		3	
Hematologic malignancy	1		2	

*Round brackets = discontinuation IPPV, invasive positive pressure ventilation; NIPPV, non-invasive positive pressure ventilation.

**Table 2:** Airway stenting group.

Case	Duration from onset (months)	Duration of stent placement (months)	Cause of death	procedures	Date of stenting (year/month)	Type of stent	Complication
1	290	87	Pneumonia	2	2006/11	Spiral Z	Granulation tissue, mucostasis
2	89	66	Respiratory failure	1	2005/6	Ultraflex	Granulation tissue, mucostasis
3	110	100	Respiratory failure	1	2007/8	Ultraflex	Granulation tissue, mucostasis
4	24	24	Respiratory failure	1	2013/11	Ultraflex	Granulation tissue, mucostasis
5	250	201	Respiratory failure	4	2014/11	Silicone, Ultraflex	Granulation tissue, mucostasis
6	45	27	Pneumonia	1	2012/12	Ultraflex	Granulation tissue
7	26	14	Respiratory failure	1	2013/11	Ultraflex	Mucostasis
8	57	41	Pneumonia	1	2013/7	Ultraflex	Granulation tissue, mucostasis
9	100	88	Respiratory failure	1	2012/12	Ultraflex	Granulation tissue mucostasis
10	143	137	Survived	3	2011/2	Ultraflex	Granulation tissue, mucostasis
11	235	235	Survived	1	2003/1	Ultraflex	Granulation tissue
12	53	21	Survived	3	2018/2, 2019/12 (removal)	Silicone	Granulation tissue
13	12	11	Hematologic malignancy	2	2008/10	Ultraflex	None

**Figure 2:**
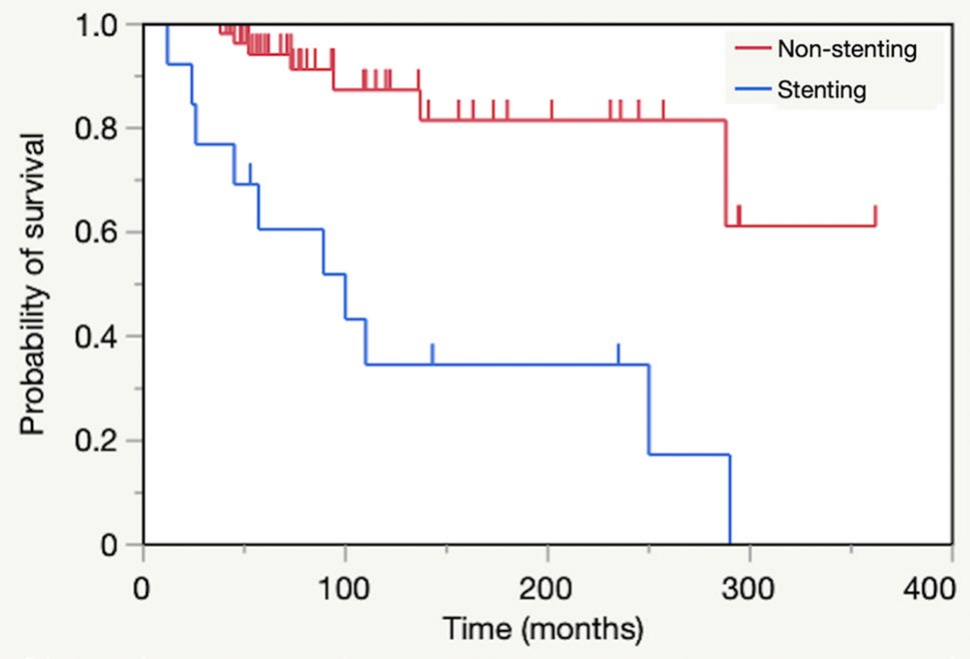
Kaplan–Meier curves for airway stenting (n = 13) and non-stenting (n = 64) groups.

Figure 2 shows survival rates for the airway stenting and non-stenting groups. Log rank test revealed that the stenting group had a significantly lower survival rate than the non-stenting group (*p* < 0.001). Of the 10 patients who died in the stenting group, 6 were due to acute respiratory failure, 3 infections, and 1 hematologic malignancy. In total, 50 airway stents were placed. Of these, 46 were Ultraflex stents, 2 spiral Z stents, and 2 silicone stents. In the two patients with silicone stents, 1 patient needed an additional Ultraflex stent 33 months after the second re-stenting due to granulation tissue, while the second patient had their silicone stent removed after 22 months. Stent-related complications were granulation tissue (85%) and mucostasis (69%).

Of the 64 patients in the non-stenting group, immunosuppressive agents and biologics were used for 83% and 59%, respectively. NIPPV was administered to 12 patients, while tracheostomy was performed in 18 patients, 6 requiring mechanical ventilation. Tracheostomy was closed successfully in 3 patients. Thirteen patients did not have airway stenosis and lower mortality rates were observed in patients without airway stenting. Of the seven patients who died, 3 were due to respiratory failure, 2 infections, and 2 from hematologic malignancy.

Patients were further divided into biologics and non-biologics groups. In the stenting group, 3 patients were administered biologics, with 2 patients continuing tocilizumab. One patient showed no response to any biologics, but Janus kinase inhibitor was effective.

In the non-stenting group, the following biologics were administered: infliximab (29), certolizumab (5), golimumab (1), tocilizumab (22), abatacept (1), and rituximab (1). Infliximab, tocilizumab and certolizumab were effective in 17 of 29, 14 of 22, and 3 of 5, respectively. Golimumab was effective in one case where the aforementioned biologics failed. Abatacept was effective in 1 patient. Supplementary Table 1 describes the biologics administered to all patients from each group.

**Figure 3:**
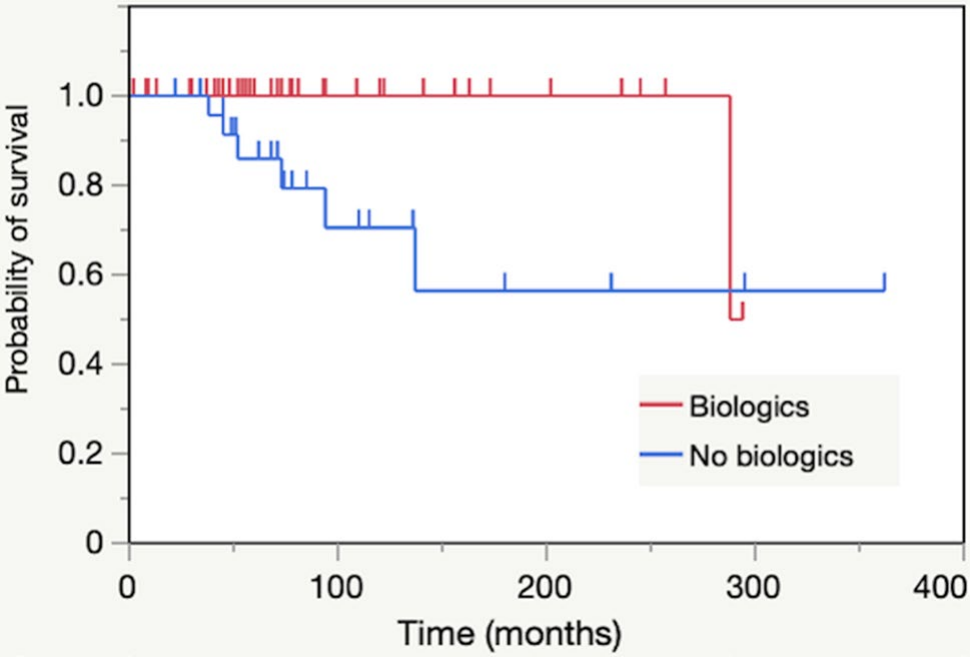
Kaplan–Meier curves for the non-stenting group with biologics (n = 39) and without biologics (n = 25).

Log rank tests revealed that the use of biologics had a significantly higher survival rate than those without (*p* = 0.014, Fig. 3). Of the 13 patients in the stenting group, 3 who received biologics survived after stenting. Six patients in the non-stenting group did not receive biologics and 1 patient with hematologic malignancy discontinued biologics for chemotherapy. All patients who were not administered or discontinued biologics died.

## Discussion

Airway involvement in RP can consist of several conditions and locations, and therapeutic interventions should be based on these circumstances. Although stenting should be considered for severe airway stenosis with malacia, stent-related complications were almost inevitable over long-term follow-up. Therefore, it is important to introduce strong pharmacotherapy as early as possible. Furthermore, tracheostomy and positive pressure ventilation should be considered without hesitation if TBM worsens. The results of this study consider that biologics might be effective in reducing airway inflammation and preventing severe airway disorders that require airway stenting.

In this study, respiratory failure, infections, and haematological malignancies were the main causes of death. Severe TBM is associated with a higher risk of respiratory failure and infection. TBM in RP is characterized by circumferential airway thickening, and if treatment is delayed, irreversible airway narrowing with malacia can occur. In addition, RP has been reported to be associated with haematological malignancies such as leukaemia, myelodysplastic syndrome, and lymphoma^9–13^. Chemotherapy for haematological malignancies can cause bone marrow suppression and respiratory infections can be life-threatening.

Survival rates for RP patients with airway involvement have been reported to be about 70%^14^. In this study, airway malacia resulted in higher mortality in both the airway stenting and non-stenting groups. All patients who underwent airway stenting developed airway malacia. The use of self-expanding metallic stents have been reported to be useful in the central airway for severe TBM in RP^6,7,15–19^. However, complications from metallic stents such as fractures, airway ruptures or restenosis due to granulation tissue and mucous, are sometimes fatal^20^. In this study, complications from metallic stents were found to be inevitable over a long period of time. As a result, the long-term survival rate was poor for the stenting group. Hence, the U.S. Food Drug Administration does not recommend metallic stents for benign central airway obstruction; instead, recommending silicone stents^21^. The placement of a silicone stent often relies on the rigid bronchoscopic procedure, but it can be difficult to insert a rigid bronchoscope through the narrowed airways of a RP patient. Furthermore, silicone stents have similar complications as metallic stents, and TBM caused by RP can extend from the main bronchus to the peripheral airways, making it difficult to cover the whole airway lesion. Since NIPPV can be used to manage TBM in RP^19–22^, it should be considered before and after stenting. When NIPPV cannot maintain airway patency, tracheostomy with mechanical ventilation should be performed. In patients without airway malacia, subglottic stenosis is a dangerous condition that can lead to choking. Tracheostomy should also be considered for subglottic stenosis without hesitation.

At present, no treatment guidelines for RP have been established. Systemic corticosteroids and immunosuppressive agents are standard medications for RP. The early administration of systemic steroid and immunosuppressive agents with airway interventions can improve the prognosis of RP patients with airway involvement. However, RP is often misdiagnosed as severe asthma, and these drugs are not always effective for RP.

Over 10 years ago, airway stenting was actively performed in patients with severe airway stenosis. However, stent-related complications inevitably occur in the long-term. Recently, the efficacy and safety of biologics have been reported for RP^23,24^. It is difficult to achieve a complete response using biologics, however TNFα inhibitors and tocilizumab are effective drugs for RP. In our institution, infliximab and tocilizumab are mainly used in RP patients with airway involvement. However, it is necessary to pay attention to the side effects when using biologics, especially infection. High-dose systemic steroids are often given during TBM exacerbations, but long-term high-dose systemic steroids are also high risk. The early administration of biologics can avoid high-dose systemic steroids and decrease steroid-related complications. In addition, the use of biologics is increasing, and the number of stenting cases is decreasing. This trend is also reflected at our institution where the implantation of metallic stents have not been performed since November 2014 due to the use of biologics. In this study, the use of biologics led to a reduction in mortality for airway involvement by RP. The early administration of biologics might have prevented the progress of airway stenosis and reduced acute exacerbations. Therefore, airway stenting could be considered as a last resort with removable silicone stents placed when possible.

There were some limitations to this study. This was a retrospective study performed at a single centre. However, as RP is a rare disease, it is difficult to identify sufficient numbers of patients to assess by a randomized control study. Furthermore, treatment options varied by doctor over the duration of this study. Another limitation was the difference in the number of cases and disease severity between stenting and non-stenting groups. In the stenting group, cases were more severe with more tracheostomies performed than in the non-stenting group. Although a selection bias is very important limitation, stenting still remains the last option treatment due to the issue of long-term complications.

In conclusion, Biologics should be administrated early in RP patients with airway involvement. If pharmacological therapies are ineffective, NIPPV should be initiated, while tracheostomy should be reserved for severe subglottic stenosis. Finally, if airway stenting is required due to unstable respiratory conditions, removable stents should be considered before metallic stents when possible. It would be desirable to establish a standard treatment protocol by accumulating more evidence for airway involvements in RP.

## Methods

This retrospective study enrolled consecutive RP patients with airway involvement from January 1, 2004, to August 30, 2022. All methods in this study were conducted in accordance with the Declaration of Helsinki and the International Conference on Harmonisation Tripartite Guideline for Good Clinical Practice. The institutional review board of St. Marianna University School of Medicine approved this study (2406), which was registered with the University Hospital Medical Information Network Clinical Trials Registry (UMIN000018937). The need for informed consent of patients was waived by the review board of St. Marianna University School of Medicine.

Clinical information was collected from medical records. The McAdam’s or Damiani and Levine diagnostic criteria were used for RP diagnosis^25,26^. Airway involvement was confirmed by chest CT or bronchoscopy. Typical CT findings showed airway thickness, calcification and narrowing on inspiration, and airway malacia and air trapping on expiration. Bronchoscopic findings were characterized by airway narrowing and dynamic airway collapse during expiration. Airway malacia was defined as paired inspiratory and expiratory CT with over a 50% reduction of the cross-sectional area at the airway lesion. Patients were divided into with and without malacia groups. RP cases were further divided into stenting and non-stenting groups. For metallic stents, Ultraflex stents (Boston Scientific, Natick, Massachusetts, USA), and Spiral Z stents (Cook Inc., Bloomington, Indiana, USA) were used, while implanted silicone stents were Dumon stents (Novatech, Grasse, France). Stenting and non-stenting groups were divided into biologics and without biologics groups.

The Kaplan–Meier method was used to calculate the survival rates and log rank tests were used to analyse biologics and non-biologics groups. Results are presented as mean ± SD, and *p* values of < 0.05 were considered statistically significant. All statistical analyses were performed using JMP software version 16 (SAS Institution, Cary, NC, USA).

## Supplementary Information

Supplementary Information.

## Data Availability

The datasets used and analysed during the current study are available from the corresponding author on reasonable request.
